# Effect of Er: YAG laser, phthalocyanine activated photodynamic therapy, and bioactive glass nanoparticles on smear layer removal and push out bond strength of quartz fiber posts to canal dentin: a SEM assessment

**DOI:** 10.3389/fdmed.2025.1665937

**Published:** 2025-10-21

**Authors:** Okba Mahmoud, Erum Zain

**Affiliations:** ^1^ Clinical Science Department, Ajman University, Ajman, United Arab Emirates; ^2^ Center of Medical and Bioallied Health Sciences Research, Ajman University, Ajman, United Arab Emirates; ^3^ Department of Restorative Dentistry & Endodontics, Faculty of Dentistry, SEGi University, Petaling Jaya, Selangor, Malaysia

**Keywords:** phthalocyanine, bioactive glass nanoparticles, smear layer, quartz fiber post, scanning electron microscope, sustainable development goals (SDG) 4 & 9

## Abstract

**Introduction:**

Teeth that have undergone endodontic treatment often suffer a considerable loss in structural strength, necessitating the insertion of a post into the root canal to maintain stability for both restoration and functionality. While NaOCl and EDTA are standard disinfectants, they come with drawbacks such as toxicity to surrounding tissues, potential allergic reactions, interference with resin sealer polymerization, and dentin erosion. Consequently, finding more effective alternatives is crucial.

**Objectives:**

Effectiveness of various contemporary post space disinfectants, i.e., Er: YAG laser, Phthalocyanine (Pc) activated Photodynamic Therapy (PDT), Bioactive glass nanoparticles (BAGNPs) on the removal of the smear layer (SL) and the push-out bond strength (PBS) of quartz fiber post (QFP) to radicular dentin.

**Methods:**

Forty-eight mature, single-rooted human mandibular premolars were selected for the study. After ensuring proper disinfection, standard root canal procedures were carried out on each tooth. Post space was prepared. The samples were randomly split into four groups based on the post-space disinfection protocols (*n* = 12). NaOCl + 17% EDTA, Er: YAG laser + 17% EDTA, *Pc*(*PDT*) 17% EDTA, and BAGNPs 17% EDTA. SL removing efficiency was evaluated using SEM on two samples from each group. Bonding of QFP was performed in the post space, followed by sectioning of the canal dentin (coronal, middle, apical). The PBS via universal testing machine and failure mode via stereomicroscopy were quantitatively evaluated. ANOVA was used to compare the results across the different groups, accompanied by the Tukey *post hoc* test *p* < 0.05.

**Results:**

Post space treated with Er: YAG_+_ EDTA presented the highest SL removal and maximum PBS. Whereas Group 4 (BAGNPs_+_EDTA) samples revealed the lowest SL removal efficacy and minimum PBS.

**Conclusion:**

Er: YAG + EDTA used as a post-space disinfectant showed significantly better results in terms of SL removal and PBS across all three sections compared to the other groups. Er: YAG + EDTA could potentially serve as an alternative to 2.5% NaOCl + EDTA.

## 1 Introduction

Teeth that have been treated endodontically often experience a significant reduction in their structural integrity, making it necessary to place a post within the root canal to ensure stability for both restoration and function (1). In today's dental field, there is an increasing emphasis on aesthetic posts and cores, which has led to the innovation of metal-free post-and-core systems, particularly those utilizing zirconium dioxide and fiber-reinforced composite (FRC) materials (2). Quartz fiber posts (QFP) offer significant benefits in dental restoration, primarily because their elastic modulus is similar to that of dentin. This similarity enhances stress distribution and reduces the likelihood of root fractures when compared to metal posts (3, 4). Additionally, QFP offers superior aesthetics and enhanced light transmission, resulting in restorations that appear more natural. However, the post space must be meticulously cleaned and free from any smear layer (SL), as this factor significantly impacts the push-out bond strength (PBS) results (5).

In the field of endodontics, sodium hypochlorite (NaOCl) is commonly considered the preferred disinfectant for root canal cleaning due to its potent antimicrobial characteristics and its ability to effectively dissolve organic tissue. Nonetheless, NaOCl affects the organic elements of the SL (6). Therefore, to enhance decontamination, ethylenediaminetetraacetic acid (EDTA) is used after NaOCl application, as it efficiently removes the remaining inorganic debris and the SL that develops on tooth surfaces, thereby improving bond strength (6). Nonetheless, both NaOCl and EDTA are associated with limitations, including toxicity to the tissues around the root, possible allergic reactions, and disruption of resin sealer polymerization as well as dentin erosion (7). Therefore, the pursuit of more effective alternatives becomes essential.

Recent advancements in technology have facilitated the implementation of laser and photodynamic therapy (PDT) across various restorative and endodontic practices (8). Considering the wavelength of 2,940 nm, Er: YAG lasers are completely absorbed in the surface of target tissues, leading to minimal thermal propagation, which is advantageous for root canal therapy (9). Furthermore, work by AlSahaf has shown that Er: YAG laser, particularly in conjunction with EDTA, efficiently removes the SL and enhances the bonding of dental materials (10). Photodynamic therapy (PDT) in the field of dentistry represents a non-invasive approach that employs light-activated photosensitizers to eradicate bacteria, fungi, and certain cancer cells (11). Its application has expanded across various areas of dentistry. Numerous photosensitizers (PSs) with distinct compositions exist; however, phthalocyanine (Pc) has garnered considerable interest due to its remarkable antibacterial efficacy and color stability, as noted by Sahin and associates (12). Nevertheless, there remains a lack of literature evidence that has illuminated their effects on SL removal and the PBS of QFP, indicating a necessity for future research.

With rapid progress in nanotechnology, nanoparticles are showing great potential in the medical field. These tiny particles are characterized by at least one dimension ranging from 1–100 nm, which makes them highly effective for various applications (13). Obeid et al. explored the antimicrobial properties of Bioactive glass nanoparticles (BAGNPs) when used as an intracanal medicament against E. faecalis, resulting in the highest rate of bacterial elimination (14). However, there is a scarcity of studies that have clarified its role as a disinfectant for post spaces and its effect on SL removal and the bond strength of QFP.

The research was conducted under the hypothesis that the differences observed between groups would not be statistically significant in the SL removal capacity when utilizing contemporary post-space disinfectants in comparison to the conventional control. Additionally, it was anticipated that the strength of the bond between QFP and post space dentin with modern disinfectants would be analogous to that achieved with NaOCl and EDTA (control). Consequently, the objectives of this study were to evaluate the effectiveness of various contemporary post space disinfectants (NaOCl+17%EDTA, Er: YAG laser+17% EDTA, Phthalocyanine-Pc activated by PDT + 17% EDTA, and BAGNPs 17% EDTA) on the removal of the SL and PBS between QFP and the post space dentin.

## 2 Materials and methods

**Preparation of BAGNPs** Commercially available “standard bioactive glass 45S5 (Perioglass™, US Biomaterials Corp., Alachua, FL, USA)” was obtained from a certified supplier (Sigma Aldrich, Berlin, Germany). In addition, “bioactive glass nanopowder (BAG-np)” was synthesized using the “sol-gel” method by “Nano-Stream” (15). To formulate the oxide composition, silicon and phosphorus alkoxides were mixed with sodium and calcium sources, specifically sodium hydroxide and calcium hydroxide. The mixture was dissolved with the help of deionized water and ethyl alcohol, serving as solvents. The gel was created at 70°C and a pH of 2, then left to age for a week, followed by heat treatment at temperatures reaching 800°C (15). The resulting particles were examined using scanning electron microscopy (Quanta FEG 250, FEI, Hillsboro, OR, USA), operating at 120 kV to verify that a particle size of less than 100 nm was obtained.

**Sample preparation:** A total of 48 mandibular premolars, each with a single root and fully developed apices, which had been extracted due to periodontal issues, were used in this study. The study was approved by the Ajman University, UAE, Ethics Committee (Project Number: D-F-H-16-Jun-2025). The present study followed the checklist for reporting *in vitro* study (CRIS) guidelines and was conducted following the Declaration of Helsinki (16). The patient, whose teeth were utilized for experimental purposes in this study, provided written consent. Using the OpenEpi sample size calculator, the sample size was calculated (17, 18). Teeth that displayed curvature of 6-25° or greater on periapical radiograph, dilacerations, and fractured roots were excluded. The samples were subjected to disinfection with a thymol solution and were thoroughly cleaned using an ultrasonic scaler (Woodpecker Ultrasonic Scaler UDS-J, Guilin, Guangxi, China) to gently clean the specimens, removing any debris or remaining soft tissue. After cleaning, the samples were kept in a 0.9% saline solution at 37℃ to preserve their condition. The teeth were decoronated using a carborundum disc at the cementoenamel junction to ensure that each specimen maintained a consistent length of 15 mm (19).

**Root canal treatment** Each root canal was meticulously instrumented using ProTaper rotary system (Dentsply Sirona, Charlotte, North Carolina), progressing to the F3 file, and a standardized irrigation protocol was carried out, which included the application of 5 ml of 2.5% NaOCl (Supply Chain Management Co., Ltd, Jinan, Shandong, China) solution followed by 5 ml of 17% EDTA (Stardent Equipment Co., Limited, Guangdong, China) as a final disinfectant for 1 min. Canals were dried using paper points (Dentsply Maillefer). Resin-based Nonacrylic AH-26 sealer (Dentsply DeTrey, Beijing, China) was applied on the canal wall using a 20 K file rotated in a counterclockwise manner. A gutta-percha (GP) master cone (Dentsply Maillefer), coated with AH-26 sealer, was subsequently condensed into the canal using an endodontic condenser and plugger. A temporary filling was placed in the access cavity. The samples were then stored in 100% humidity at 37°C for 48 h. After this incubation period, each root was embedded securely into a cylindrical mold made from self-curing acrylic resin. The post-space preparation began 48 h after completing the root canal treatment (20).

**Post space preparation** Gutta-percha was carefully removed from the upper part of the canals using Peeso reamers sizes #2 and #3 (MANI Inc.). The post space was prepared to a depth of 9 mm below the cemental junction using a low-speed drill #3, as recommended by the manufacturer. The root canal treatment and post space preparation were performed by an experienced endodontist.

### 2.1 Group allocation

The samples were randomly assigned to four different groups, each following a specific disinfection protocol (*n* = 12).

#### 2.1.1 Group 1 (NaOCl + EDTA)

The samples were rinsed using 5 ml of 2.5% NaOCl for 30 s and air dried. The samples were then treated with 17% EDTA for 30 s to aid in cleaning and SL removal from post space, using a 27-G irrigating tip (Endo-Eze; Ultradent, South Jordan, UT) (6).

#### 2.1.2 Group 2 (Er: YAG laser + 17% EDTA)

Er: YAG laser (Lightwalker; Fotona, Ljubljana, Slovenia) was used to activate the irrigating solution at a wavelength of 2,940 nm. H14 handpiece and a conical PIPS fiber tip (600/9) was carefully positioned at the canal orifice. The Er: YAG laser was set on Super Short Pulse (SSP) with power 0.3W, frequency 15 Hz, and 20 MJ with no air and water spray. The fiber tip was kept perpendicular, steady, and at a constant distance to activate 2 ml of 3% sodium hypochlorite (NaOCl) refreshed continuously every 30 s. This process was repeated three times with 30 s pauses in between. The post-space was dried. The final step involved applying 17% EDTA for 30 s to complete the procedure (21).

#### 2.1.3 Group 3 (phthalocyanine-Pc 17% EDTA)

The post space was carefully filled with 1 ml of a 6 micromolar (µM) solution of Pc (Santa Cruz Biotechnology, Inc., Santa Cruz, CA) and left undisturbed for 5 min to allow for adequate interaction with the post space. Light-emitting diode (LED) Fotosan 630 (CMS Dental, Seoul, Korea) wavelength of 620–640 nm (85%) with a peak of 630 nm, and an intensity of 2–4 mW/cm^2^ to irradiate the post space for 60 s (22). Following the procedure, Pc was aspirated, and the post-space was dried. The final step involved applying 17% EDTA for 30 s to complete the procedure.

#### 2.1.4 Group 4 (BAGNPs 17% EDTA)

The BAGNPs 1% solution was prepared by dissolving 1 g of BAGNPs into 100 ml of distilled water. The prepared 1% BAGNP solution (0.5 ml) was injected into the post space using an irrigating syringe and left for 1 min to take effect. The solution of BAGNPs was aspirated, and the post-space was dried. The final step involved applying 17% EDTA for 30 s to complete the procedure (23).

### 2.2 SL removal assessment

Once the intracanal treatments were completed, two teeth from each group were sectioned into two halves. This was done by carefully creating two longitudinal grooves on each tooth using a diamond-coated high-speed bur, taking special care not to breach the canal itself. The teeth were then split along the grooves using a mallet and chisel, with the help of a modified cementum spatula, producing mesial and distal halves of the root canal. Each half was then gold-plated with a 15–20 nm gold-palladium layer and observed using SEM (Quanta FEG 250, FEI, Hillsboro, OR, USA). During the experiment, the resolution achieved was approximately 40 nm. The evaluation of smear layer removal quality was conducted by two independent experts through the analysis of photographs acquired after scanning electron microscopy (SEM), utilizing a scoring system ranging from 1–5 as proposed by Hulsmann et al. (24). The criteria were established as follows:

Score 1: Complete removal of SL, with exposure of dentinal tubules (DT).
Score 2: Minimal SL present over the canal, with many DT observable.
Score 3: Presence of SL and debris obscuring the canal walls, with a limited number of DT visualized.
Score 4: Root canal surface entirely covered by SL DT, not visualized.
Score 5: Significant SL and debris obscuring the surface of the root canal.

Two independent endodontist specialists, blinded by the experimental groups, assessed the SL kappa score (0.78).

### 2.3 Bonding of quartz fiber post

QFP (RTD ILLUSION #3; St. Egreve, France) underwent a cleaning process that involved the application of 70% ethanol and then gently air-drying. Self-adhesive dual-cure resin cement (Maxcem Elite, BisCem, RelyX Unicem) was utilized to secure the posts in 10 teeth from each group. The methods for applying the cement were executed in line with the manufacturer's recommendations, a Lentulo spiral was used for the application process. Subsequently, the posts were gently placed to their full depth within the prepared cavities, applying light finger pressure for a few seconds. Excess luting material was carefully removed with a fine brush to maintain precision. To complete the process, the luting agent was cured by exposing it to light polymerization, with the LED light positioned directly at the coronal ends of the posts. The specimens, after preparation, were kept in distilled water to maintain hydration at a temperature of 37°C for 24 h before testing (25). Before the sectioning, samples were thermocycled (GeneBio System, Inc., Toronto, Canada), between temperatures of 5℃–55℃ for 15 s each bath for a total of 5,000 cycles.

### 2.4 Sectioning of the samples

The specimens were meticulously sectioned utilizing an Isomet butcher saw (Logix, Technova Noida, Gautam Buddha Nagar, Uttar Pradesh, India). The posts were positioned at a 90-degree angle to the root's longitudinal axis, ensuring proper alignment, while water cooling was utilized to mitigate thermal effects. From each specimen, three sections of 2 mm in thickness were obtained from each third of the root (coronal, middle, apical).

### 2.5 PBS testing

The PBS was quantitatively evaluated utilizing a universal testing machine (UTM) (EMIC DL 2,000; Sao Jose dos Pinhais, PR, Brazil) by exerting a force at a velocity of 0.5 mm/min. The posts were pushed in the apical-to-coronal direction to ensure they were moved toward the wider part of the root slice, helping to avoid any taper limitations during the procedure until the segment of the quartz post that had been relined was detached from the root slice. To articulate the bond strength in megapascals (MPa), the force at which failure occurred, measured in newtons, was divided by the surface area (mm^2^) of the post-dentin interface (26).

**Fracture pattern assessment.** The specimen failure mode was determined using a stereomicroscope (Leica, MZ125, Milton Keynes, UK) at x40 magnification. The fractured surfaces were classified as adhesive, cohesive, and admixed (27).

To assess failure types, cohesive, adhesive, or admixed, two independent endodontist specialists, blinded by the experimental groups, assessed the type of failure, using Kappa statistics (0.81).

**Statistical analysis.** The normality of the data distribution was evaluated through the application of the Kolmogorov–Smirnov test. Data analysis was conducted utilizing one-way ANOVA, supplemented by the Tukey *post hoc* test. All analytical procedures were performed utilizing SPSS software version 17 (SPSS Inc., Chicago, USA). The significance level of *p* < 0.05 was established.

## 3 Results

### 3.1 SL removal assessment

**Figure 1:**
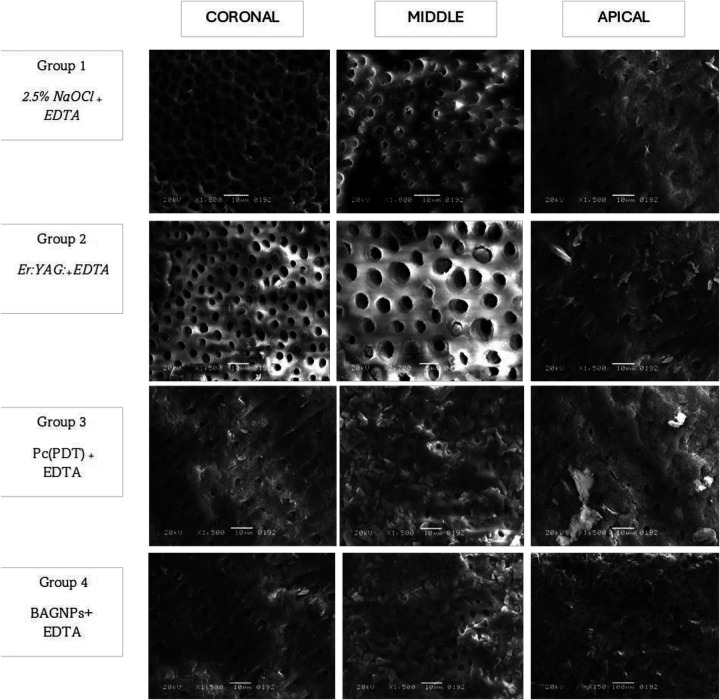
SEM micrograph of canal wall irrigated with 2.5% NaOCl + EDTA. The SEM image shows that dentinal tubules are open, and the SL has been partially removed in the coronal, middle, and apical thirds of the dentin. Areas of debris and smear layer are observable. Canal dentin irrigated with Er: YAG: +EDTA pronounced open dentinal tubules. Radicular canal irrigated with PC + EDTA and BAGNP + EDTA SEM images captured indicate the presence of SL and debris that covered the walls, revealing only a few dentinal tubules observed open in all three thirds (coronal, middle, and Apical).

SL removal from the post space after using different post space disinfection protocols is displayed in Table 1. The cervical third of Group 2 (Er: YAG:_+_ EDTA) treated teeth presented the highest SL removal (1.54 ± 0.06). Whereas, the apical third of Group 4 (BAGNPs_+_ EDTA) samples revealed the lowest SL removal (3.93 ± 0.72). Comparison among different tested groups exhibited that SL removal in Group 3 [Pc (PDT)_+_EDTA] (Cervical: 2.77 ± 0.23, Middle: 3.21 ± 0.28, and Apical: 3.84 ± 0.41) and Group 4 (Cervical: 2.69 ± 0.20, Middle: 3.12 ± 0.22, and Apical: 3.93 ± 0.72) presented comparable scores at all three-thirds of the canal (*p* > 0.05). However, Group 1 (2.5% NaOCl _+_ EDTA) (Cervical: 2.11 ± 0.12, middle: 2.17 ± 0.04, apical: 2.84 ± 0.44) and Group 2 treated teeth (Cervical: 1.54 ± 0.06, middle: 1.58 ± 0.07, and apical: 2.56 ± 0.81) presented significant difference from each other and other tested groups *(p* *<* *0.05)* (Figure 1).

**Table 1:** SL removal efficacy from post space after the application of various disinfection protocols.

Tested groups	Mean ± SD Cervical	Mean ± SD Middle	Mean ± SD Apical	*p*-value!
Group 1: 2.5% NaOCl _+_ EDTA	2.11 ± 0.12^a,A^	2.17 ± 0.04^a,A^	2.84 ± 0.44^a,B^	*p* < 0.05
Group 2: Er:YAG:_+_ EDTA	1.54 ± 0.06^b,A^	1.58 ± 0.07^b,A^	2.56 ± 0.81^b,B^	
Group 3: Pc(PDT)_+_ EDTA	2.77 ± 0.23^c,A^	3.21 ± 0.28^c,A^	3.84 ± 0.41^c,B^	
Group 4: BAGNPs + EDTA	2.69 ± 0.20^c,A^	3.12 ± 0.22^c,A^	3.93 ± 0.72^c,B^	

! ANOVA.

NaOCl, sodium hypochlorite; EDTA, ethylenediamine-tetraacetic acid; BAGNPs, bioactive glass nanoparticles, Pc, phthalocyanine; PDT, photodynamic therapy.

Different superscript lower-case letters within the same column signify statistically significant differences (*p* < 0.05), *post hoc* Tukey.

Data accompanied by varying upper-case letters within each respective row indicate significant disparities (*p* < 0.05), *post hoc* Tukey.

Intra-group comparison reveals that smear removal efficacy was found to be comparable between the coronal and middle third in all groups (*p* > 0.05). However, the efficacy of SL was found to be significantly higher in the apical third in all groups (*p* < 0.05).

### 3.2 PBS testing

The PBS of QFP after using different post-space disinfection protocols is displayed in Table 2. The cervical third of Group 2 (Er: YAG + EDTA) treated teeth presented the highest PBS (10.41 ± 0.21 MPa). Whereas, the apical third of Group 4 (BAGNPs + EDTA) samples revealed the lowest PBS (7.21 ± 0.52 MPa). Comparison among different tested groups exhibited that Group 3 [Pc (PDT) + EDTA] (Cervical: 8.47 ± 0.09 MPa, Middle: 8.54 ± 0.32 MPa, and Apical: 7.43 ± 0.47 MPa) and Group 4 (Cervical: 8.39 ± 0.08 MPa, Middle: 8.48 ± 0.41 MPa, and Apical: 7.21 ± 0.52 MPa) disinfected canals presented comparable PBS at all three thirds (*p* > 0.05). However, Group 1 (2.5% NaOCl + EDTA) (Cervical: 9.16 ± 0.17 MPa, middle: 9.07 ± 0.12 MPa, apical: 8.54 ± 0.24 MPa) and Group 2 treated teeth (Cervical: 10.41 ± 0.21 MPa, middle: 10.39 ± 0. MPa, and apical: 9.89 ± 0.32 MPa) presented significant differences from each other and other tested groups (*p* *<* *0.05*).

**Table 2:** PBS of QFP after using different post-space disinfection protocols.

Tested groups	Mean ± SD Cervical	Mean ± SD Middle	Mean ± SD Apical	*p*-value!
Group 1: 2.5% NaOCl _+_ EDTA	9.16 ± 0.17^a,A^	9.07 ± 0.12^a,A^	8.54 ± 0.24^a,B^	*p* < 0.05
Group 2: Er:YAG:_+_ EDTA	10.41 ± 0.21^b,A^	10.39 ± 0.25^b,A^	9.89 ± 0.32^b,B^	
Group 3: Pc (PDT) + EDTA	8.47 ± 0.09^c,A^	8.54 ± 0.32^c,A^	7.43 ± 0.47^c,B^	
Group 4: BAGNPs _+_ EDTA	8.39 ± 0.08^c,A^	8.48 ± 0.41^c,A^	7.21 ± 0.52^c,B^	

! ANOVA.

NaOCl, sodium hypochlorite; EDTA, ethylenediamine-tetraacetic acid; BAGNPs, bioactive glass nanoparticles; Pc, Phthalocyanine; PDT, photodynamic therapy.

Different superscript lower-case letters within the identical column signify a statistically significant difference (*p* < 0.05), *post hoc* Tukey.

Upper-case letters within each respective row indicate significant disparities (*p* < 0.05), *post hoc* Tukey.

An intra-group comparison reveals that PBS was found to be comparable between the coronal and middle third in all groups (*p* > 0.05). However, the PBS was found to be significantly lower in the apical third in all groups (*p* < 0.05).

### 3.3 Failure mode assessment

**Figure 2:**
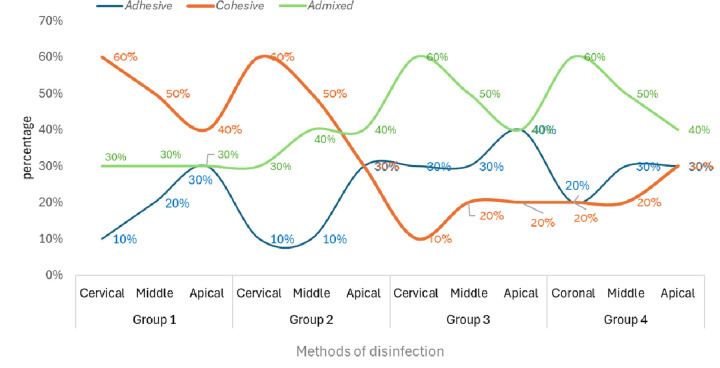
Percentage of failure modes in each investigated group.

Figure 2 displays the percentage of failure modes for each investigated group. Groups 1 and 2 presented cohesive failures the most. Whereas Groups 3 and 4 exhibited admixed failures predominantly.

## 4 Discussion

The present investigation was predicated on the assertion that there would be no statistically significant difference in the SL removal efficacy when utilizing contemporary post-space disinfectants, Er: YAG laser, PC (PDT), BAGNP, in combination with EDTA as a final disinfectant, as opposed to the conventional method (2.5% NaOCl + EDTA). Furthermore, it was also hypothesized that the bond strength of QFP, when affixed to post-space following contemporary post-space disinfection, would be comparable to that obtained with NaOCl and EDTA. Considering the results of the present investigation, it was observed that the proposed hypothesis was entirely rejected, as the experimental group Er: YAG + EDTA showed significantly higher bond integrity and SL removal efficacy, while Pc(PDT) and BAGNP exhibited significantly lower and comparable bond strength values and SL removal efficacy compared to the control.

Based on the existing indexed academic literature, it can be asserted that the SL, which constitutes a delicate film of debris adhering to the walls of the root canal post-instrumentation, typically diminishes the bond strength of both posts and sealing materials (28, 29). Er: YAG laser pretreated root canal presented the highest scores of SL removal and PBS of QFP with the root canal dentin. Er: YAG laser is particularly effective on hard tissues because its 2,940 nm wavelength is readily absorbed by both hydroxyapatite and water (30). This enables it to effectively cut and remove dental hard tissues like dentin and enamel, often eliminating the need for local anesthetics (31). Additionally, combining the Er: YAG laser with EDTA has been shown to enhance the removal of the SL, creating better conditions for bonding restorative materials (32). The Er: YAG laser operates on the principle that its energy is absorbed by water molecules in the SL, leading to rapid vaporization and tissue removal (33). This process effectively removes the SL from the canal wall. Lab-based analysis by Celiksoz and coworkers has provided evidence that Er: YAG laser treatment, particularly in conjunction with EDTA, is effective in the removal of SL and significantly boosts the adhesion properties of dental materials, which justifies the outcomes of the existing study (34). However, some researchers have reported contradictory outcomes and revealed that the Er: YAG laser produces melted and sealed dentinal tubules, which eventually decreases the bond strength. This is following the findings of ex vivo analysis conducted by Alsahhaf and coworkers (11). Different laser parameters (frequency, power, power density), structure (human/bovine dentin and enamel), and material type may have contributed to differences in outcomes.

The satisfactory performance of NaOCL and EDTA is in agreement with the outcomes of lab-based analysis conducted by Aljamhan and colleagues (35) as well as Alkhudhairy et al (36). The oxidative characteristics of NaOCl solution lead to the degradation of dentinal collagen and interfere with the polymerization process of bonding cement (37). Conversely, EDTA eliminates the oxidative layer, enhancing the surface's capacity to adhere to resin cement, which in turn boosts bond strength (38). In this study, a combination of 2.5% NaOCl and EDTA was employed to achieve a synergistic effect. This effect is further corroborated by SEM images, which reveal that the majority of dentinal tubules are open and SL has been eliminated in the coronal and middle sections of the dentin.

BAGNPs and PC-PDT-treated canals exhibited the least and comparable results in terms of SL removal and bond integrity compared to other comparative groups. This marks the first study to acknowledge the role of BAGNPs as a root canal irrigant in the removal of SL and PBS of QFP. In an earlier study, conducted by Sadooq and colleagues, it was found that BAGNPs remarkably display antibacterial properties (39). Nonetheless, in this study, BAGNPs demonstrate limited effectiveness in eliminating SL. According to the author, the absence of proteolytic activity, lack of chelating abilities, and issues related to particle size might impede its effectiveness in SL removal. In the present study, PC activated by PDT was utilized as a photosensitizer because of its excellent absorption in the phototherapeutic range, remarkable light stability, significant phototoxicity, and minimal dark toxicity, along with its efficiency in generating singlet oxygen (22). However, PC-PDT in the present study demonstrated low SL removal efficacy, and PBS. This outcome can be attributed to the lower hydrophilicity of PC-PDT, which negatively hindered PBS and poor SL removal efficacy (22). Both types of post-space disinfection (PC-PDT, BAGNPs), the outcome can be confirmed by the SEM image showing the presence of SL and debris that covered the walls and orifice opening of DT, revealing only a few dentinal tubules observed to be open.

The intragroup comparison revealed that across all the examined groups, the apical segment exhibited markedly lower SL removal and PBS of QFP. This phenomenon can be attributed to the fact that the apical area of the canal contains complexities such as variations in curvature, canal dimensions, taper, diameter, ramifications, deltas, isthmuses, and the permeability of dentin (40). Concerning the failure mode, it was noted that samples in groups 1 and 2 demonstrated cohesive failure type in abundance. Cohesive failures are attributed to adhesive system issues, C-factor stresses, sclerotic dentin, and air bubble incorporation (41). Whereas, an adhesive failure pattern is observed due to incomplete smear layer removal, poor adhesive penetration, and incomplete polymerization (42).

The current investigation reveals several inherent constraints. This study is defined by an ex vivo methodology; therefore, it is crucial to conduct extensive longitudinal clinical research to accurately ascertain the desirable characteristics of the newest post-space irrigants that are being utilized. In addition, the use of laser and photodynamic therapy (PDT) was performed with singular parameters; hence, alternative parameters must be assessed too. The absence of advanced surface analysis techniques like Raman spectroscopy and x-ray photoelectron spectroscopy (XPS) could restrict a more detailed understanding of the groups that were tested. It is essential to evaluate the antibacterial effectiveness of these modern canal disinfectants against E. *faecalis*, as this bacterium is the leading cause of root canal infections and treatment failures.

## 5 Conclusion

In the study, the application of Er: YAG + EDTA laser for post-space disinfection achieved superior results in Smear layer removal and push-out bond strength compared to all other groups examined. These results indicate that Er: YAG + EDTA can be effectively used as an alternative to conventional canal irrigation techniques when bonding a quartz post to canal dentin.

## Data Availability

The raw data supporting the conclusions of this article will be made available by the authors, without undue reservation.
